# Silver-109/Silver/Gold Nanoparticle-Enhanced Target Surface-Assisted Laser Desorption/Ionisation Mass Spectrometry—The New Methods for an Assessment of Mycotoxin Concentration on Building Materials

**DOI:** 10.3390/toxins13010045

**Published:** 2021-01-09

**Authors:** Justyna Szulc, Artur Kołodziej, Tomasz Ruman

**Affiliations:** 1 Department of Environmental Biotechnology, Lodz University of Technology, Wólczańska 171/173, Łódź 90-924, Poland; 2 Doctoral School of Engineering and Technical Sciences at the Rzeszow University of Technology, 8 Powstańców Warszawy Ave., 35-959 Rzeszów, Poland; d497@stud.prz.edu.pl; 3 Faculty of Chemistry, Rzeszów University of Technology, 6 Powstańców Warszawy Ave., 35-959 Rzeszów, Poland; tomruman@prz.edu.pl

**Keywords:** mycotoxins, building materials, laser desorption/ionisation mass spectrometry, metabolomics method, nanoparticles

## Abstract

This study aimed to detect and quantify mycotoxins on building materials using innovative laser mass spectroscopy methods—silver-109/silver/gold nanoparticle-enhanced target surface-assisted laser desorption/ionisation mass spectrometry (^109^AgNPs, AgNPs and AuNPs SALDI). Results from SALDI-type methods were also compared with commonly used matrix-assisted laser desorption/ionization (MALDI) mass spectrometry. Standards of seven moulds mycotoxin in a final concentration of 100 µg/mL for patulin, citrinin, 3-nitropropionic acid, alternariol and 20 µg/mL for sterigmatocystin, cyclopiazonic acid, roquefortine C in the mixture were tested in pure solutions and after extraction from the plasterboards. Among the studied SALDI-type method, the lowest detection limits and the highest signal intensity of the mycotoxins tested were obtained with the use of ^109^AgNPs SALDI MS. The ^109^AgNPs method may be considered as an alternative to the currently most frequently used method MALDI MS and also liquid chromatography tandem mass spectrometry LC-MS/MS for mycotoxin determination. Future studies should attempt to use these methods for mass spectrometry imaging (MSI) to evaluate spatial distribution and depth of mycotoxin penetration into building materials.

## 1. Introduction

Mycotoxins (approximately 400, low-molecular-weight substances, produced by more than 200 mould species), are compounds exhibiting a great structural diversity as well as chemical and thermal stability [1,2]. In the literature, the greatest attention is paid to the occurrence of toxic mould’s compounds in food products and feed. Mycotoxins may pose a potential threat to human and animal health, and exposure to them may result in acute and chronic diseases [3,4]. However, there has been a growing body of scientific literature indicating that exposure to mycotoxins and toxigenic moulds adversely affect the health of people working and living in water-damaged buildings (homes, offices, schools and public facilities) [5]. Dampness and moulds growth on building materials occurs in 10–50% of buildings located in Europe, Asia, Australia, and North America [6,7]. Fungi, including moulds from genera *Alternaria* (*A. alternata*)*, Aspergillus* (*A. flavus, A. niger* and *A. versicolor*), *Penicillium* (*P. aurantiogriseum, P. chrysogenum, P. expansum, and P. viridicatum*), *Stachybotrys* (*S. chartarum*) and many others, are able to grow on all common types of building materials including wallpaper, plasterboards, wooden elements, emulsion paints, materials for insulation, and finishing [6,8,9,10].

People staying in mouldy buildings have an increased risk of airways infections and other health problems including asthma, and respiratory symptoms [11,12]. Recently a new term, dampness, and mould hypersensitivity syndrome (DMHS), was introduced for disease previously described as biotoxin-related illness, mould-related illness, and chronic inflammatory response syndrome etc. [5,13,14].

It was proven that mycotoxins inhalation might be even ten times more toxic than other routes of entry (via ingestion and skin); therefore, the emission of mycotoxins into the air from building materials is dangerous [15]. The most frequently identified mycotoxins on building materials are trichothecenes, ochratoxin A, sterigmatocystin. Mycotoxins have also been detected in dust from ventilation systems and in the indoor air where residents have complained of health problems potentially caused by the presence of moulds in the buildings [16,17,18,19].

Although the problem of the presence of mycotoxins on building materials is not new, its study still poses many difficulties. Among mould genera such us *Alternaria, Aspergillus, Penicillium,* only a few experienced microbiologists are able to identify environmental isolates to the level of species [20,21]. It should also be noted that many inappropriate sequences (from misidentified strains) can be found, as well as misidentified mycotoxins from a wide variety of mould species which can be found in databases [22]. Moreover, unidentified and masked mycotoxins and synergistic effects of metabolites can be discovered in the extracts of fungi, which have been detailed and analysed for metabolites production [7,21,23].

Immunochemical-based and chromatographic-based methods are two groups of the methods for mycotoxins analysis [4,24]. Enzyme-linked immunoassay (ELISA) is primarily used for the detection of mycotoxins in cereals. Aflatoxin B1, the deoxynivalenol (DON), ochratoxin A and zearalenone were determined by ELISA. Monoclonal antibodies have also been developed for the diacetoxyscirpentriol, 3- and 15-acetyl-DON, sporidesmin A, roridin, and other mycotoxins [25].

Nowadays the liquid chromatography with tandem mass spectrometry (MS/MS) methods, including multi-mycotoxin methods, are accurate, highly selective and sensitive methods of mycotoxin determination. The aim of the LC-MS/MS multi-mycotoxin methods is to avoid cumbersome and complicated cleaning methods and the use of many analytical techniques [1,2,26,27]. Multi-mycotoxin methods are validated for many types of matrices i.e., cereals, spices, fruit, oilseed and others plant and food products [26,28,29].

An alternative for LC-MS/MS methods in the determination of secondary metabolites of fungi are the soft-ionisation, laser-based family of techniques—laser desorption/ionisation mass spectrometry (LDI MS) methods including matrix-assisted laser desorption/ionisation (MALDI) and surface-assisted laser desorption/ionisation (SALDI).

MALDI analyses of the medium–high molecular mass of compounds (mainly proteins and lipids) are a valuable tool in identification of species of fungi with clinical significance [30,31]. However, classical MALDI is characterized by some limitations which include: a high level of chemical background (in the low-mass region—*m*/*z* 1000), the effect of the sweet spot and in the case of low-polarity compounds, low ionisation efficiency. These disadvantages do not occur in laser desorption–ionisation methods that use a steel target covered with silver or gold nanoparticles instead of the matrix, i.e., the surface-assisted laser desorption ionisation method (SALDI) such as silver-109/silver/gold nanoparticle-enhanced target surface-assisted laser desorption/ionisation mass spectrometry, respectively: ^109^AgNPET, AgNPET, and AuNPET SALDI methods [32,33].

Kuo et al., (2017) used Langmuir–Blodgett films with silver nanocrystals (cube, cuboctahedron and octahedron) for glucose detection in SALDI MS. The authors obtained significantly improved parameters of analysis (the intensity of the signal, signal-to-noise ratio, noise reproducibility) compared to MALDI with α-cyano-4-hydroxycinnamic acid and 2,5-dihydroxybenzoic acid matrices [34]. AuNPET (gold nanoparticle-enhanced target) and gold nanoparticle multilayers (AuNPs-ML) were used recently for mass spectral analysis of compounds with different polarities, over a wide range of *m*/*z* values. Amino acids, glycosides, lipids, saccharides, nucleosidesand others were successfully studied using these methods [35,36,37]. Pan et al. (2019) used gold nanoparticles in form of two-dimensional multilayered AuNPs thin film (MTF-AuNPs) for direct analysis of bone biomarker (hydroxyproline) in the research of osteoporosis detection [38]. SALDI AuNPET, in contrast with conventional MALDI, allows the analysis of low molecular weight compounds. The other advantages include a reduction in the background level, a high signal-to-noise (S/N) ratio, and a precise internal spectral calibration [35,36].

In the recent studies, SALDI methods were used for c analysis of metabolites of microbial origin in objects of cultural heritage. AuNPET SALDI allows the detection of microbial metabolites, including tocopherols, 2-methyl-6-phytylquinol and 3-hydroxy-L-kynurenine, which may be responsible for foxing formation [39].

The ^109^AgNPET allows the discovery of mycotoxins (aflatoxins B1, and B2, ochratoxin B andT2-toxin), and organic acids on the surfaces of historical photographs [40]. Szulc et al. (2020) using the same method proved the presence of *Aspergillus* and *Penicillium* metabolites including micotoxins and their derivatives (e.g., aflatoxin M2, 3-*O*-methylviridicatin, dihydroaflatoxin G1 and dihydrohydroxyaflatoxin B1) and antibiotics (penicillin K) in beeswax seal samples [41].

Based on the above, the suitability of NPET SALDI with silver and gold nanoparticles methods in mycotoxins analysis should be further investigated.

This study aimed to detect and quantify mycotoxins for building materials using innovative laser mass spectroscopy methods—silver/silver-109/gold nanoparticle-enhanced target surface-assisted laser desorption/ionisation mass spectrometry.

## 2. Results

The presented data (Table 1) contain the first presentation of detection limits for mycotoxin detection using silver, silver-109 and gold nanoparticle-enhanced targets used in surface-assisted laser desorption/ionisation mass spectrometry. The commonly used MALDI and LC-MS/MS method quoted different detection limits, depending on the matrix. Still, foodstuffs were the most commonly employed products (np. apple puree, hazelnut, sweetcorn, and green pepper) with the limits of detection (LOD) ranging from 0.2 to 134.6 µg/kg [1,26,27]. Tuomi et al. (2000) analysed 79 samples of mouldy building materials, mainly interior finishes containing paper, wood and plastic, (e.g., wallpaper, plywood, plasterboards, mineral wool) and others. The authors obtained the LOD value for sterigmatocistin and citrinin amounting to 2 and 20 ng, respectively [42]. Concerning different methods of LOD and and lower limits of quantification (LLOQ) presentation in the literature and a very limited scope of studies involving building materials, it is hard to compare the obtained results.

**Figure 1:**
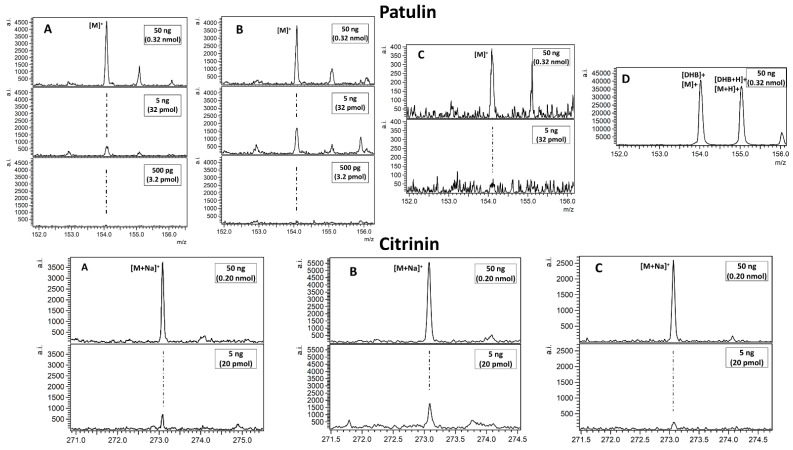
Spectra presenting laser desorption/ionisation mass spectrometry (LDI MS) obtained results on different targets ((**A**) ^109^AgNPET; (**B**) AgNPET; (**C**) AuNPET; (**D**) MALDI with the 2,5-dihydroxybenzoic acid (DHB) matrix) for patulin and citrinin. The spectra panel contains a magnified view on most intense adduct signals. Text in boxes provides information on a compound amount per spot.

**Figure 2:**
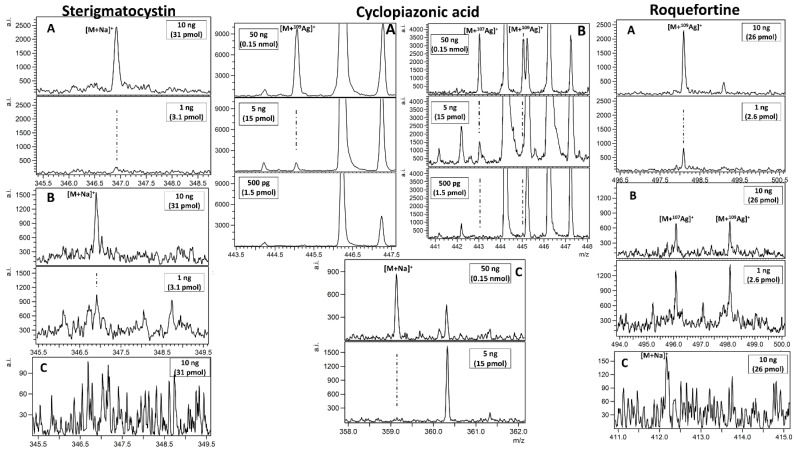
Spectra presenting LDI MS obtained results on different targets ((**A**) ^109^AgNPET; (**B**) AgNPET; (C) AuNPET; (**D**) MALDI with the DHB matrix) for sterigmatocystin, cyclopiazonic acid and roquefortine C. The spectra panel contains a magnified view of the most intense adduct signals. Text in boxes provides information on a compound amount per spot.

**Figure 3:**
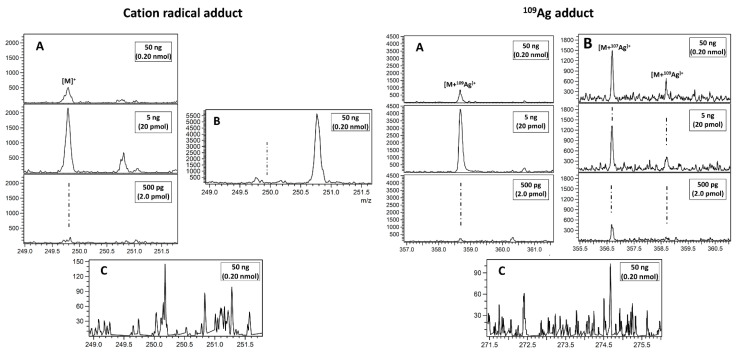
Spectra presenting LDI MS obtained results for citrinin after extraction from plasterboard ((**A**) ^109^AgNPET; (**B**) AgNPET; (**C**) AuNPET; (**D**) MALDI with the DHB matrix). Text in boxes provides information on a compound amount per spot.

**Figure 4:**
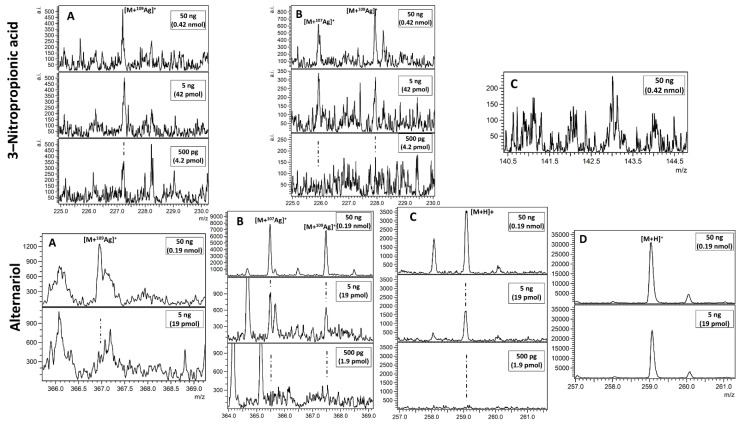
Spectra presenting LDI MS obtained results of 3-nitropropionic acid and alternariol on (**A**) ^109^AgNPET, (**B**) AgNPET and (**C**) AuNPET; (**D**) MALDI with the DHB matrix. Insets contain a magnified view of the most intense adduct signals. Text in boxes provides information on a compound amount per spot.

**Figure 5:**
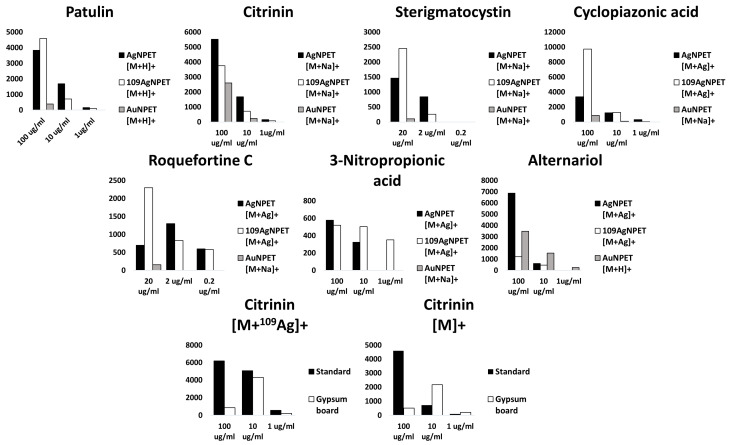
Column charts presenting intensity values for the most intense ions of the studied compounds for given concentrations. The colour of the bar represents the target type (black—AgNPET, grey—AuNPET, white—^109^AgNPET). Two charts (bottom) present intensity values comparison for citrinin standard measured with the use of ^109^AgNPET measured directly (black) and after extraction from plasterboard.

MALDI mass spectrometry is commonly used for biomolecule detection as shown by the very large amount of publications in last fifteen years. In case of mycotoxin mixtures studied in this work, only alternariol was found as a proton adduct (Figure 4D). The possible explanation is that relatively poor, linear internal calibration performed on two signals emitted by the 2,5-dihydroxybenzoic acid (DHB) matrix is producing too many *m*/*z* matching errors over 5 ppm forcing us to reject possible peak assignment. This kind of problem is virtually absent with our methods based on nanoparticles, which, after laser pulse irradiation, are emitting a series of well-defined ions used for enhanced cubic calibration. Examples of spectra presenting LDI MS-obtained results on different SALDI for tested mycotoxins are given in Figure 1, Figure 2, Figure 3 and Figure 4. All the studied compounds were found in LDI MS spectra in the form of adducts, mostly with metal ions, which is expected behaviour for compounds of medium-to-low polarity. The most intense ions found for citrinin, sterigmatocystin, cyclopiazonic acid, roquefortine C, 3-nitropropionic acid and alternariol were silver adducts. Much more polar patulin was found mostly in the form of proton adduct for all targets: ^109^AgNPET, AgNPET and AuNPET. The observed very high affinity of the studied compounds toward silver ions suggests strongly that the methods used typically for ionisation of these compounds—electrospray ionisation and MALDI are not the best choices as the studied ions are usually proton adducts. Intensity values on different MALDI-type targets for different concentrations of mycotoxins standards are presented in Figure 5. Intensity values depend on mycotoxin type and its concentration for particular MALDI-type targets. The highest intensity in the case of each tested standard concentration was obtained for AgNPET for citrinin. For patulin, sterigmatocystin, cyclopiazonic acid and roquefortine, the greatest intensity was obtained for the highest tested concentrations using ^109^AgNPET; the greater intensity characterised the AgNPET method in the case of lower standard concentrations. Comparing the MALDI type targets, the lowest intensities were obtained with the use of gold nanoparticles, and the highest for silver nanoparticles and ^109^AgNPs.

The results obtained for 3-nitropropionic acid and alternariol are very interesting. Intensive signals (300–500) were obtained for the first compound only in the ^109^AgNPET method. The AuNPET method provided far better results in the alternariol analysis for all tested concentrations.

The intensity values for three concentrations of mycotoxin standards (100 µg/mL, 10 µg/mL and 1 µg/mL), tested with three methods—^109^AgNPs, AgNPs and AuNPs—are shown in Figure 5. The intensity was demonstrated to depend on the concentration of the identified compound and the target type. The highest intensity for the majority of the tested compounds was achieved for identification with the ^109^AgNPs method, including but not limited to 100 µg/mL concentration. Such a situation was observed for patulin, sterigmatocistin, cyclopiazonic acid and roquefortine C. The AgNPs method rendered the best intensity for each concentration of citrinin. This method turned out to be most effective also for 100 µg/mL 3-nitropropionic acid and alternariol. The lowest concentrations of the two compounds were identified with the highest intensity owing to ^109^NPs and AuNPs, respectively. The lowest intensities for all tested mycotoxins were obtained in the AuNPs method, except for alternariol, which rendered the lowest signals for ^109^NPs.

Figure 5 shows the change in the intensity of mycotoxins tested as diluted standards, and after extraction from plasterboards. Among the seven mycotoxins tested, only citrinin (cation radical adduct) was successfully determined from plasterboard.

The mycotoxin tested directly and not extracted from the building material revealed the highest intensity in each tested concentration for ^109^Ag adduct of citrinin. The higher the concentration of the identified compound, the greater the difference in the signal intensity was. A slightly different situation was demonstrated for the cation radical adduct of citrinin. In this case, the standard solution revealed greater intensity for 100 µg/mL concentration, whereas a higher intensity for two lower concentrations was discovered for the extracts obtained from plasterboards.

Due to the unlimited number of types and composition of building materials and the common presence of additional contamination (dust), from an analytical point of view, building materials are a challenging “matrix”. Moreover, the variety of fungal metabolites makes mycotoxin analysis on building materials a difficult task. The SALDI methods, including but not limited to ^109^AgNPs, presented in this paper, may be a good alternative for MALDI MS and the LC-MS/MS method currently most popular for mycotoxin identification. They are less time-consuming (measurement time of ca. 15 s/sample; alternative LC-MS/MS—from 15 to 45 min/sample) and the whole measurement procedure is automated. However, the routine use of ^109^AgNPs SALDI MS in mycotoxin analysis on building materials requires further research aimed at verification and validation of this method.

Neither the LC/MS/MS nor any other method used currently for mycotoxin analysis offers the opportunity to determine the spatial distribution/depth of mycotoxin penetration into the building material. Laser Desorption/Ionisation mass spectrometry allows the imaging of metabolites and an analysis of their distribution in the tested samples.

Szulc et al. (2017) used the high-resolution surface assisted laser desorption/ionisation time-of-flight mass spectrometry-based on a gold nanoparticle-enhanced target (AuNPET SALDI-ToF-MS) imaging method in order to assess the metabolite profile of the selected mould species under model conditions on microbial medium and plasterboard [7]. The authors detected compounds in the range of 80–1950 *m*/*z* and identified 89 metabolites from 48 metabolomic pathways in wated extracts from building materials. Moreover, they presented the changes in the amount and types of identified compounds as a result of various growth conditions (type of growth media, single culture or mixed population) [7].

Therefore, future studies should aim at the utilisation of such laser spectrometry methods as ^109^AgNPET/Ag/AuNPET SALDI MSI to detect mycotoxins and determine the degree of the building material contamination with the mycotoxins, through an in-situ assessment of the spatial distribution (environmental sample with an increase in the toxigenic mould). The effectiveness of the methods should be verified by analysing the extracts obtained from other building materials susceptible to contamination with mycotoxins, e.g., wood, wallpaper, and paint coats.

## 3. Conclusions

Among the Laser Desorption/Ionisation mass spectrometry methods tested in the present study, the highest overall applicability and universality, as well as the lowest detection limits of the tested mycotoxins were found for ^109^AgNPET. Other methods such as AgNPET and AuNPET SALDI MS had higher LOD and LLOQ limits. Nanoparticle-based methods were compared with commonly used MALDI MS which gave very small positive detections due to inferior calibration. The ^109^AgNPs method, following by verification and validation, may provide an alternative to the currently most frequently used LC-MS/MS method for mycotoxin determination. Future studies should attempt to use these methods for MSI imaging to evaluate spatial distribution and depth of mycotoxin penetration into building materials.

## 4. Materials and Methods

### 4.1. Preparation of ^109^AgNPET Target

An amount of 10 mg of silver-109 trifluoroacetate was dissolved in anhydrous, tetrahydrofurane (volume of 50 mL) and the solution was poured into a beaker with laser target plate (stainless steel target of 35 × 45 × 0.8 mm size). Solid 2,5-dihydroxybenzoic acid (35 mg) was added and the solution was left for 24 h. The target plate was then washed with tetrahydrofurane, acetone and deionised water (three times), gently cleaned with a cotton wool ball, again washed 3 times with tetrahydrofurane, and then with water and acetonitrile. The ^109^AgNPET target was characterized by Nizioł and Ruman (2013) [43].

### 4.2. Preparation of AgNPET

Silver trifluoroacetate (AgTFA, 40 mg) was dissolved in anhydrous tetrahydrofurane (volume of 50 mL) and the solution was poured into a beaker containing a laser target plate (stainless steel target of 35 × 45 × 0.8 mm size). Solid 2,5-dihydroxybenzoic acid (80 mg) was added and solution was left for 24 h. The rest of the procedure—cleaning of target—is the same as for ^109^AgNPET preparation. The AgNPET target was characterized by Nizioł et al. (2013) [44].

### 4.3. Preparation of AuNPET

Chloro(trimethylophosphite)gold(I) complex (amount of 8 mg) was dissolved in 15 mL of acetonitrile, and then the solution was poured into a beaker containing a target plate (stainless steel target of 35 × 45 × 0.8 mm size). The pyridine–borane complex (volume of 52 μL) was added with the use of a gas-tight syringe and, following stirring, the solution was left for 24 h. The target plate was then washed with acetonitrile and deionised water (three times), then it very gentle cleaned with a cotton wool ball and again 3-times with acetonitrile and water. AuNPET target was characterized by Sekuła et al. (2015) [35].

### 4.4. Moulds Mycotoxins Standards Analysis

Standards of seven moulds mycotoxin (patulin, citrinin, 3-nitropropionic acid, alternariol sterigmatocystin, cyclopiazonic acid, roquefortine C) were purchase from Sigma-Aldrich (Poznań, Poland). Standards were weighed and dissolved in acetonitrile to give a final concentration of 100 µg/mL for patulin, citrinin, 3-nitropropionic acid, alternariol and 20 µg/mL for sterigmatocystin, cyclopiazonic acid, roquefortine C in the mixture. Lower concentration solutions were prepared by diluting ten times higher concentration ones. A volume of 0.5 µL of mycotoxins solution was placed directly on 109AgNPET, AgNPET and AuNPET plate, air-dried, and the targets were inserted into MS apparatus for measurement.

MALDI experiments were performed using DHB matrix solution (saturated matrix in acetonitrile with 0.5% of trifluoroacetic acid) were made by the drying droplet method (1:1 vol/vol matrix:sample solution).

### 4.5. Analysis of Mycotoxin Standards on Plasterboards

Unimpregnated plasterboard with a 9.5 mm thickness and A2 class of fire resistance (Siniat, Warszawa, Poland) was purchase from retail sales. Sample sizes of 0.5 × 1 cm were cut from the plasterboard. Volumes of 100 µL of the mycotoxin solutions (contains all tested mycotoxins, see Section 4.4) were applied to the samples. After 30 min, the plasterboard fragments were transferred to test tubes, and 2 mL of acetonitrile were added to each sample. Samples were homogenised manually, and a volume of 1.5 mL solution was collected from each sample and transferred into new Eppendorf tubes. The collected solutions were centrifuged (6000 rpm, 1 min) and the supernatants (1.4 mL) were left to dry under a high vacuum. After drying, acetonitrile was added to each sample to obtain the same concentration of mycotoxins in the sample as in the standard solutions. A volume of 0.5 μL of the mixture was placed directly on 109AgNPET and AuNPET plate, air-dried and targets were inserted into the MS apparatus for measurements. MALDI MS measurements were performed as stated in the previous Section 4.4.

### 4.6. LDI Mass Spectrometry

LDI-ToF mass spectrometry experiments were performed in the reflectron mode on Bruker Autoflex Speed ToF mass spectrometer (Bruker Autoflex Speed, Bruker Daltonics, Bremen, Germeny) equipped with a SmartBeam II laser (355 nm). The laser impulse energy was in range of 100–150 μJ, laser repetition rate 1 kHz. The total number of laser shots was 4000 for each sample spot. The mentioned amount of laser shots was divided into four points symmetrically placed around the spot centre. At each point, 1000 laser shots were performed with a default random walk (random points with 50 laser shots). The measurement range was *m*/*z* 80–2000. Suppression was on for ions of *m*/*z* lower than 80. The first accelerating voltage was held at 19 kV, and the second ion source voltage at 16.7 kV. The reflector voltages were 21 kV (the first) and 9.55 kV (the second). The data were calibrated and analysed with FlexAnalysis (version 3.3, Bruker Daltonics Leipzig, Germany) using centroid calibration model. The mass calibration for ^109^AgNPET used was enhanced cubic calibration typically based on at least five calibration points and it was made using internal standards which were silver-109 ions and cluster from ^109^Ag^+^ to ^109^Ag_9_^+^; for AgNPET calibration was made using all isotopic peaks from Ag^+^ to Ag_5_^+^; for AuNPET was made using gold ions and cluster from Au^+^ to Au_5_^+^. MALDI spectra were calibrated on DHB ions: [M + H] ^+^ (*m*/*z* 155.03444) and [2M + H] ^+^ (*m*/*z* 309.06105). All quantification data were made based on triplicate experiments. Data in Table 1 present average intensities from triplicate experiments. All spectra were baseline corrected and recalibrated in FlexAnalysis 3.3. For estimation of LOD and LLOQ, linear regression for S/N ratios of 3 and 5, respectively, was used. Regression was based on spectral data of the lowest concentration sample with detectable analyte signal.

## Figures and Tables

**Table 1:** Limits of detection and lower limits of quantification for studied mycotoxins.

Toxin	^109^AgNPs		AgNPs		AuNPs		MALDI	
	LOD ^a^ (ng/mL) (µM)	LLOQ ^b^ (ng/mL)	LOD ^a^ (ng/mL) (µM)	LLOQ ^b^ (ng/mL)	LOD ^a^ (ng/mL) (µM)	LLOQ ^b^ (ng/mL)	LOD ^a^ (ng/mL) (µM)	LLOQ ^b^ (ng/mL)
Patulin	4271 27.7	7118	5565 36.1	9276	30,000 194.7	50,000	nd	nd
Citrinin	6830 27.3	11,384	6905 27.6	11,508	5405 21.6	9009	nd	nd
Citrinin (Cation Radical Adduct) Extracted from Plasterboards	561 2.24	935	nd	nd	96,774 248.5	161,290	nd	nd
Citrinin (^109^Ag Adduct) Extracted from PlasterBoards	396 1.58	660	58,824 151.0	98,039	nd	nd	nd	nd
Sterigmatocystin	15,272 47.1	25,453	19,990 61.6	33,317	nd	nd	nd	nd
Cyclopiazonic Acid	2756 8.19	4594	17,298 51.4	28,830	17,143 51.0	28,571	nd	nd
Roquefortine C	7935 20.4	13,225	27,883 71.6	46,471	93,750 240.7	156,250	nd	nd
Alternariol	8092 31.3	13,487	5367 13.8	8945	3834 14.8	6390	1939 4.98	3232
3-Nitropropionic Acid	4088 34.3	6814	62,500 160.5	104,167	nd	nd	nd	nd

^a^ based on a signal-to-noise (S/N) ratio of 3; ^b^ based on an S/N ratio of 5; LOD—limit of detection; LLOQ—lower limit of quantification; “nd”—signal not detected.

## Data Availability

Data sharing not applicable.
